# Effect of nanoemulsified and encapsulated *Planiliza abu* protein on fortified yogurt

**DOI:** 10.1002/fsn3.4150

**Published:** 2024-11-12

**Authors:** Nasrin Vakili, Maryam Ataee, Shapour Kakoolaki, Hamed Ahari, Arman Ghorbanzade

**Affiliations:** ^1^ Department of Food Hygiene, Science and Research Branch Islamic Azad University Tehran Iran; ^2^ Iranian Fisheries Science Research Institute, Agriculture Research Education and Extension Organization (AREEO) Tehran Iran; ^3^ Department of Food Science and Technology, Science and Research Branch Islamic Azad University Tehran Iran; ^4^ Department of Aquatic Health and Disease, Veterinary Science Faculty Islamic Azad University Tehran Iran

**Keywords:** amino acid enrichment, fish‐based protein, protein enrichment, protein hydrolysates, yogurt

## Abstract

Fortified dairy products such as yogurt have attracted a lot of attention due to the increasing concern for public health. This study aimed to determine the effects of nanoemulsified and a microencapsulated protein hydrolysate obtained from *Planiliza abu* on some of the properties of yogurt. The physicochemical, rheological, microbiological, and sensory evaluation of the fortified yogurt samples stored at 4°C were assessed during 21 days. The fish protein hydrolysates (FPHs) < or >10 kDa protein were used. The amino acid profile of *Planiliza abu* was glutamic acid (78.99%), aspartic acid (59.53%), and lysine (53.54%). No significant change was observed in titratable acidity (TA) and pH of supplemented yogurts during refrigeration. The highest survival level of lactic acid bacteria (LAB), water‐holding capacity (WHC), and viscosity and the lowest syneresis were observed in fortified samples with FPH <10 kDa. The highest ferric reducing antioxidant power (FRAP) was achieved in yogurt fortified with nanoemulsion FPH < 10 kDa (NEh10a, 0.41 mg/g). The results showed that fortified yogurt with FPH, particularly less than 10 kDa, is among the desirable functional food with appropriate gel network and consistency as well as better taste and mouth feel, and higher overall acceptance.

## Graphical abstract

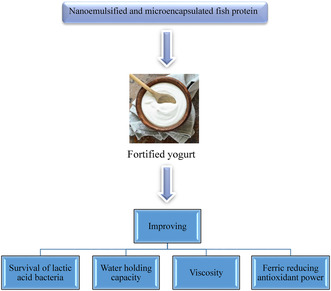

## 1 INTRODUCTION

Nowadays, there is an increasing demand for healthy and functional foods with bioactive compounds globally (Liu et al., 2019). The worldwide market value of functional foods is predicted to rise from 168 billion dollars in 2013 to more than 300 billion dollars in 2020 (Gumus & Gharibzahedi, 2021). Among these beneficial foods, fermented dairy products, mainly yogurt, broadly used throughout the world, present health profits derived from lactic acid bacteria (LAB) (Lima et al., 2021; Ma et al., 2019). Recently, bioactive peptides taken from food proteins such as have been noticed more because of their multiple biological properties and beneficial health effects (Honkanen, 2009). In a study, the antiglycation potential of fish, maize, and whey protein hydrolysates was investigated and the highest inhibitory effect was observed in fish protein hydrolysate about 98% inhibition (Arasteh et al., 2023). A few studies, concerning adding bioactive components to yogurt such as grape seed protein hydrolysate (Varedesara et al., 2021), stripped weakfish protein hydrolysate (Lima et al., 2021), sturgeon gelatin hydrolysates (Gheshlaghi et al., 2021), wheat germ protein hydrolysates (Ghelich et al., 2022), and protein hydrolysate from Hyrcanian goby (Rezaei, Noori, et al., 2020). Fish protein hydrolysates (FPHs) presented as nanoemulsion or microencapsulated would be protected that can act as a barrier between the oxidative compounds and FPH, resulting in a delayed reaction (Almasi et al., 2021; Benimana et al., 2022; Ghorbanzade et al., 2017; Zeashan et al., 2020). The emulsification or microencapsulation process would improve the food's palatability for consumers (Gharibzahedi & Jafari, 2017). Encapsulation also increases the texture of the yogurt (Afzaal et al., 2018; Yousefi et al., 2022).

Consistently, proteins taken from the fish hydrolyzing process signify a really motivating source of bioactive peptides (Hemker et al., 2020). The FPH is a source of proteins with several biocapacities, such as antioxidant and radical scavenging attributes, as well as antihypertensive activities (Neves et al., 2017). Peptides with lower molecular weight, which are by‐products of protein hydrolysis, are more easily digestible, and available, and generally have more bioactivity than indigenous proteins (Karami & Akbari‐Adergani, 2019). Yogurt is a familiar ancient food that can be strengthened with bioactive compounds, particularly with fish protein (Lima et al., 2021). Among the fishes of Iran, *Planiliza abu* is a commercial affordable species (Jorfipour et al., 2022) and is used as the source of fish protein hydrolysis for the first time in this study. Enzymatic hydrolysis of fish protein, breaking the peptide bonds, has no negative impact on the amino acids profile (Karami & Akbari‐Adergani, 2019; Ma et al., 2019).

The purpose of the present research is to develop a functional yogurt through compounding a nanoemulsion and a microencapsulated protein hydrolysate from *Planiliza abu* in fortified yogurts for the first time. Consequently, the effect of the FPH on the bioactivity, rheological, physicochemical, and sensory properties of the yogurt samples after 1, 7, 14, and 21 days of cold storage was evaluated.

## 2 MATERIALS AND METHODS

### 2.1 Materials

Fresh *Planiliza abu* of 30–50 g/fish size were purchased from a local protein market, transferred to the IAU laboratory closed to ice, washed with tap water, gutting, eviscerated, and filleted. They were ground and reserved at −18°C until further use. Flavourzyme® 1000‐L enzyme was used in the hydrolysis process purchased from Sigma‐Aldrich (Germany).

### 2.2 Experimental design

Optimization of the conditions to produce fish protein hydrolysates was conducted using a three‐factor central composite rotatable design (CCRD). A rotatable design is one where the prediction variance has the same value at any two locations that are the same distance from the design center.

In this section, the experimental steps from the beginning of material preparation to yogurt production are explained in detail and all the experiments were done with three replications.

#### 2.2.1 Preparation of protein hydrolysate

The grind models were melted in a fridge (4°C) for 24 h, then mixed with isopropanol solvent at a ratio of 1:2 (*w*/*v*), and then heated for 30 min at 70°C. After evaporation of the solvent, the models were washed with distilled water (1:5, *v*/*v*) and centrifuged at 4°C for 15 min at 3000 *g*. The supernatant was removed and combined with distilled water as well as homogenized at 18928 g for 1 min. The resultant was buffered with a mixture of 0.1 M sodium citrate and 0.2 M sodium dihydrogen phosphate at a ratio of 1 to 1 (*v/w*) and then incubated at 50°C for 10 min. For enzymatic hydrolysis, a commercial aminopeptidase, Flavourzyme® 1000 L applied at 1% concentration for an hour up to 60% degrees of hydrolysis. The product was then heated using a water bath for 15 min at 90°C to deactivate the enzyme. Finally, the obtained mixture was centrifuged at 4°C for 10 min at 2000 *g*. The supernatant was collected, and 1 s of it was passed through a 10‐kDa filter to obtain peptides less than 10 kDa (Lima et al., 2021). Both portions of peptides were dried and powdered using a freeze dryer (Labconco, FreeZone 2.5 L, USA).

#### 2.2.2 Preparation of nanoemulsified fish protein hydrolysates

Nanoemulsions of FPH were prepared using the method of spontaneous formation of oil in water. For this purpose, first, a 3‐g solution of alginate in 100‐mL water was prepared by mixing sodium alginate at medium viscosity with distilled water using a magnetic stirrer. After that, it was autoclaved for 15 min at 121°C and cooled at room temperature and eventually, the prepared sterile sodium alginate solution was mixed with a suspension of less than 10 kDa‐hydrolyzed proteins with a magnetic stirrer for 20 min at 350 rpm. The afore‐mentioned suspension was formed with 5 mL of sterile peptone water 1% (w/v) and the resultant nanoemulsions were dried, and powdered with the help of a freeze dryer next to the procedure of Zhou et al. (2022) with small change. The nanoemulsion‐FPH yogurt samples are described in Table 1.

**TABLE 1:** Formulations of yogurt samples.

Treatment	Formulation
C	Control yogurt without FPH
MC	CaCl_2_‐microencapsulated yogurt
Mh10C	CaCl_2_‐microencapsulated‐FPH yogurt containing less than 10 kDa protein
MhC	CaCl_2_‐microencapsulated‐FPH yogurt containing more than 10 kDa protein
Nea	Alg‐Na nanoemulsion Yogurts
NEh10a	Alg‐Na nanoemulsion‐FPH yogurt containing less than 10 kDa protein
Neha	Alg‐Na nanoemulsion‐FPH yogurt containing more than 10 kDa protein

Abbreviations: NEh10a, Alg‐Na nanoemulsion‐FPH yogurt containing less than 10 kDa protein; NEha, Alg‐Na nanoemulsion‐FPH yogurt containing more than 10 kDa protein.

#### 2.2.3 Microencapsulation of fish protein hydrolysates

First, the mixture of 500‐mL vegetable oil and 1 g of tween 80 (0.2%) was prepared and then sodium alginate solution in water and suspension of less than 10 kDa‐hydrolyzed proteins were poured into a beaker containing the mixture. This emulsion was mixed with a magnetic stirrer at 350 rpm for 20 min. The solution of 0.1 M calcium chloride was dropwise added to the mixture to break the emulsion. The outcome was mixed at 200 rpm for 20 min. The calcium alginate capsules were separated from the aqueous phase with the separating funnel and centrifuged for 10 min at 350 *g*. The capsules were washed with distilled water and 5% glycerol to remove the remaining oil. The obtained microcapsules were dried and powdered using a freeze dryer (Talebzadeh et al., 2014). The microencapsulated FPH yogurt samples are described in Table 1.

#### 2.2.4 Yogurt preparation

The pasteurized whole milk (3% fat), whose dry matter was set at 10% from Tehran, Iran, was used in this study. The milk temperature reached 45°C using a lab‐scale viscobator. Fish protein hydrolyzed microencapsulation and nanoemulsion were then added to the plastic glasses containing milk based on the treatment plan given in Table 1. Applying a homogenizer at 65°C, the mixture was homogenized. The homogenized milk was exposed to heat at 95°C for 5 min. They were placed in a cold‐water bath until the temperature reached 43°C. The milk (100 mL) was inoculated with a yogurt starter culture (2% composed of *Streptococcus thermophilus* and *Lactobacillus bulgaricus*) from Chr. Hansen (Horsholm, Denmark), which had already been prepared and fermented until the acidity reached to pH of 4.6. Yogurts were designed in seven groups in triplicate (Table 1). After that, the models were quickly cooled to 4°C and refrigerated until the test (Massoud et al., 2015).

### 2.3 Assessment of amino acid of FPH

For determination of the amino acids, samples were subjected to acid hydrolysis prior to precolumn derivatization using phenylisothiocyanate and reverse‐phase HPLC analysis. Free amino acid composition was determined by analysis of FPH without prior acid hydrolysis.

### 2.4 Microbial suspension of lactic acid bacteria (LAB)

Lactic acid bacteria (composed of *Streptococcus thermophilus* and *Lactobacillus bulgaricus*), donated from IAU, was initially placed in the MRS broth culture medium and incubated for 24 h at 30°C. It was centrifuged for 5 min at 5000 *g* and the supernatant was then discarded, and the residue was washed with 0.1 M phosphate buffer (pH = 7). After draining the supernatant, the sediment was brought to half McFarland's turbidity with phosphate buffer, and 1 cc of the prepared solution (starter) was added to each plastic glass containing 100 ccs of the milk (Rezaei, Yeganeh, et al., 2020).

### 2.5 Evaluation of physicochemical of the yogurt

#### 2.5.1 Total titratable acidity and pH measurement

The 0.5 mL of phenolphthalein (5%) as an indicator was added to each yogurt sample (10 g) at 25°C and immediately titrated with 0.1 M sodium hydroxide solution to reveal the faint pink color that will and stable for 2 min (Eze et al., 2021). The TA was calculated using Equation 1.

(1) $$\text{Titratable acidity}\;\left(\%\right)=\frac{\mathrm{Qty}\;\text{of NaoH}\;\left(\mathrm{mL}\right)}{\mathrm{Qty}\;\text{of sample}\;\left(g\right)}\times 0.009\times 100.$$

The pH of 18‐h past yogurt samples stored refrigerated is gauged using a digital lab‐scale pH meter (Precisa, Switzerland). (Bondia‐Pons et al., 2007).

#### 2.5.2 Syneresis and viscosity

The viscosity (expressed in cPs) of the 18‐h past yogurt examples kept at refrigeration was measured by applying a viscometer (Brookfield Engineering Laboratories, Inc., Middleboro, MA, USA) using Spindle 64. Three dial readings were measured at 1‐min intervals at 10 rpm, and their mean values were then reported.

Syneresis of the yogurt examples was measured according to the description presented by Pourbaba et al. (2021) with some changes. We centrifuged the yogurt sample of each group and replicated (20 *g*) for 30 min at 4°C at 450 rpm (Sigma 3‐18KHS, Germany). We weighed the resultant supernatant as gram (*s*), and Equation (2) was applied to eventually compute the syneresis.

(2) $$\text{Syneresis}\;\left(\%\right)=\left(s/20\;g\right)\times 100.$$

#### 2.5.3 Water‐holding capacity (WHC)

Five‐gram yogurt sample was weighed and centrifuged at 2800 g for 5 min. After separating the phases, the solid phase was collected and weighed. The capacity of the samples in holding water was measured using the following Equation (3) and reported it as a percentage (Atallah et al., 2020).

(3) $$\mathrm{WHC}=\left(W-Y\right)/W\times 100,$$

where W = weight of sample (g), Y = solid phase (g).

### 2.6 Evaluation of microbial profile

#### 2.6.1 Total plate count (TPC) and coliform count (CC)

The TPC and the CC were carried out through pour‐plating 1 mL of the yogurt sample in the Petri plate containing 12–15 mL of PCA, that is, plate count agar and violet red bile lactose (VRBL) agar (Merck, Germany) by incubating upside down at 30°C for 72 h and at 37°C for 48 h. The distinctly visible colonies were then enumerated and reported (Ghalem & Zouaoui, 2013).

#### 2.6.2 Yeast and mold count (YMC)

The YMC was measured by the pour plate method and 1 mL of the yogurt samples of each group was added in triplicate on APDA, that is, acidified potato dextrose agar medium (containing 0.01% oxytetracycline hydrochloride and 0.01% chloramphenicol). It was then incubated for 5 days at 25°C and outcomes were represented as mold, and yeast count per gram of yogurt (Kiros et al., 2016).

### 2.7 Antioxidant activities of the yogurt

#### 2.7.1 DPPH radical scavenging activity

The modified method of the 2, 2‐diphenyl‐1‐picrylhydrazyl (DPPH) radical scavenging activity was used to gauge yogurt samples' antioxidant activity (Farvin et al., 2014). For this purpose, 0.02 mL of each yogurt sample was diluted in 0.08 mL of water. It was then treated with 0.1 mL of 100 μM DPPH. The resultant aqueous was shaken and incubated for 10 min in a dark room. The absorbance by spectrophotometric method at 517 nm was calculated by the following Equation (4):

(4) $$\text{DPPH radical scavenging activity}\;\left(\%\right)=1-\frac{\text{Absorbance of sample}}{\text{Absorbance of control}}\times 100.$$

#### 2.7.2 Ferric reducing antioxidant power (FRAP) assay

The FRAP evaluation was tested by applying the OxiSelect™ Ferric Reducing Antioxidant Power (FRAP) Assay Kit (CELL BIOLABS INC., USA). The FRAP evaluation measured the antioxidant potential in the yogurt samples based on the formation of the Fe^2+^ from the reduction of the Fe^3+^ ion by antioxidant exists in the yogurt (Lee et al., 2021). When this reduction was performed, a change of color in the solution to intense blue was observed, and the absorbance was then calorimetrically measured. Accordingly, the FRAP solution was made at 55°C for 12 min following the method presented by the manufacturer. A 50 μL of each yogurt sample in triplicate was added into each microplate's well, mixed with 950 μL of prefilled FRAP solution, then incubated for 30 min at 25°C. Absorbance was then evaluated at 540–600 nm, and the FRAP property was measured by applying a Fe^2+^ standard curve.

### 2.8 Sensory evaluation

The sensory features of the samples of yogurt, including taste, odor, mouth feel, color, and overall acceptance, were performed with 14 highly trained panelists (five women and five men) based on the 5‐point hedonic procedure. The characteristics were rated with an enhancing format from 0 (highly disliked) to 4 (highly liked). Each yogurt sample was coded randomly using two‐digit numbers. Twenty‐one aliquots of yogurt (8 mL each) were taken as samples from various groups and served in 20‐mL plastic pots to each panelist in three meetings. The average scores of sensory features were applied to see the reaction of the panelists.

### 2.9 Statistical analysis

A two‐way factorial design, with sampling days (four levels) and different yogurts (seven levels) as the main impact, was implemented to evaluate the impacts on the microbial growth physicochemical properties and sensory attributes of yogurts. The Bonferroni, multiple comparison test, was used with a significance level of *p* = .05. Calculations were performed using the statistical software SPSS version 20.0.

## 3 RESULTS

### 3.1 Amino acid measurement

**FIGURE 1:**
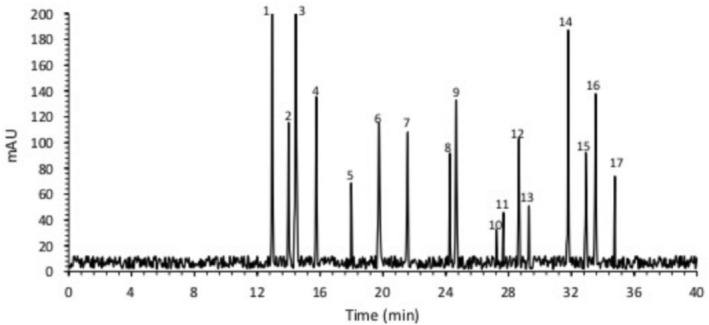
Amino acid profile of *Planiliza abu* (peaks: 1, aspartate; 2, serine; 3, glutamate; 4, glycine; 5, histidine; 6, arginine; 7, threonine; 8, proline; 9, alanine; 10, cystine; 11, tyrosine; 12, valine, 13, methionine; 14, lysine; 15, isoleucine; 16, leucine; 17, phenylalanine).

According to Table 2 and Figure 1, *Planiliza abu* is rich in aspartic acid, lysine, leucine, alanine, glutamic acid, glycine, serine, arginine, valine, threonine, proline, and phenylalanine. The amount of cysteine, tyrosine, and methionine, respectively, 9.29, 13.13, and 14.54 g/kg samples are lower in comparison with the other amino acids. Since Tryptophan is devastated through acid hydrolysis, the data on this amino acid are not in the outcome.

**TABLE 2:** Amino acid profile of *Planiliza abu.*

Peak IDs	RT (min)	Concentration (g/kg)
Aspartic acid	12.94	59.532
Serine	14.01	32.98652
Glutamic acid	14.46	78.99231
Glycine	15.77	38.68972
Histidine	17.98	19.62974
Arginine	19.75	32.90686
Threonine	21.58	30.6806
Proline	24.27	26.20825
Alanine	24.65	37.73695
Cystine	27.22	9.2934
Tyrosine	27.67	13.13032
Valine	28.66	29.43585
Methionine	29.30	14.54767
Lysine	31.80	53.54371
Isoleucine	32.92	26.35249
Leucine	33.54	39.38038
Phenylalanine	34.76	21.15362

The quality of the food protein was measured according to the 10 essential amino acids, including leucine, arginine, isoleucine, methionine, lysine, threonine, phenylalanine, tyrosine, valine, and cysteine.

### 3.2 Physicochemical analysis of the yogurt

#### 3.2.1 Titratable acidity (TA), pH, and lactic acid bacteria (LAB)

Table 3 shows the outcomes of the two‐way ANOVA. It demonstrates the effect of different fortified yogurts on pH and titratable acidity (g/100 g acetic acid). Antioxidant criteria at each sampling day were significant (*p* < .05) sometimes even as the main effect (not given in the result). The interaction effect on some properties of the samples of yogurt is shown in Table 3.

**TABLE 3:** The between‐subject effects of two‐way ANOVA test for interaction effect of independent variables for different fortified yogurts and sampling days concerning the selected criteria of samples.

Row	Effect	P. E.Sq	*F*	Sig.
1	pH	0.998	1579.50	.000
2	Titratable acidity (%)	0.999	259.57	.000
3	LAB	0.991	357.03	.000

Abbreviations: LAB, Lactic acid bacteria; P.E.Sq, Partial Eta square.

Data regarding pH and TA under differently treated yogurts in cold keeping are depicted in Table 4. The pH was 4.51–4.62 at the beginning of refrigeration for all yogurt samples (*p* > .05).

**TABLE 4:** Estimated marginal means of titratable acidity (%) and pH of the prepared yogurt models through cold storage.

	d	C	MC	Mh10C	MhC	NEa	NEh10a	NEha
pH	1	4.62 ± 0.005^a^	4.57 ± 0.005^a^	4.54 ± 0.005^a^	4.56 ± 0.005^a^	4.61 ± 0.005^a^	4.51 ± 0.005^a^	4.52 ± 0.005^a^
	7	4.39 ± 0.005^bA^	4.36 ± 0.005^bA^	4.34 ± 0.005^bA^	4.35 ± 0.005^bA^	5.38 ± 0.005^bB^	4.36 ± 0.005^bA^	4.35 ± 0.005^bA^
	14	4.20 ± 0.005^c^	4.21 ± 0.005^c^	4.22 ± 0.005^c^	4.21 ± 0.005^c^	4.20 ± 0.005^c^	4.18 ± 0.005^c^	4.19 ± 0.005^c^
	21	3.80 ± 0.005^dA^	3.89 ± 0.05^dAB^	3.97 ± 0.005^dB^	3.92 ± 0.005^dB^	3.82 ± 0.005^dA^	4.03 ± 0.005^dB^	4.01 ± 0.005^dB^
TA	1	0.43 ± 0.007^a^	0.43 ± 0.003^a^	0.45 ± 0.006^a^	0.44 ± 0.006^a^	0.43 ± 0.000^a^	0.46 ± 0.003^a^	0.47 ± 0.007^a^
	7	0.49 ± 0.003^bA^	0.62 ± 0.009^bA^	0.62 ± 0.007^bA^	0.60 ± 0.007^bA^	0.48 ± 0.003^bA^	0.64 ± 0.007^bA^	0.64 ± 0.000^bA^
	14	0.55 ± 0.000^cA^	0.65 ± 0.003^cA^	0.66 ± 0.000^cA^	0.66 ± 0.000^cA^	0.63 ± 0.003^cA^	0.78 ± 0.012^cB^	0.74 ± 0.000^cAB^
	21	1.16 ± 0.014^dA^	1.12 ± 0.022^dA^	1.01 ± 0.000^dB^	1.08 ± 0.00^dB^	1.14 ± 0.012^dA^	0.96 ± 0.007^dB^	1.00 ± 0.000^dB^

*Note*: The differences between the means with different letters in the same column are significant (*p* < 0.05).

Abbreviations: C, control; d, Days; MC, CaCl_2_‐Microencapsules Yogurts; Mh10C, CaCl_2_‐microencapsulated‐FPH yogurt containing less than 10 kDa protein; MhC, CaCl_2_‐Microencapsulated‐FPH yogurt containing more than 10 kDa protein; NEa, Alg‐Na nanoemulsion Yogurts; NEh10a, Alg‐Na nanoemulsion‐FPH yogurt containing less than 10 kDa protein; NEha, Alg‐Na nanoemulsion‐FPH yogurt containing more than 10 kDa protein.; TA, total acidity.

The pH decreased 3.80–3.97 in the cold storage time‐dependent manner, except for the Alginate‐NE group (NEa, 5.38) on day 7 but fell after that, same as other groups. The least and the utmost pH of the fortified samples were observed in NEa (3.82; no significance with control, *p* > .05) and NEh10a (4.03; only effective with NEa and control, *p* < .05) after 21 days of cold storage. Contrarily, the expected rise in TA was observed in a time‐dependent manner throughout the refrigerated storage. However, it ranged from 0.43% to 1.16% for all sample yogurts while their initial TAs were 0.43%–0.47% (*p* > .05). Exception with NEa at the minimum value (0.48%, Table 4), TA of other fortified yogurt samples reached 0.6% after 7 days, showing a meaningful variation (*p* < .05) in comparison with the control. Some changes were observed in the TA of yogurts after 14 days so that the yogurts fortified with nanoemulsion‐FPH showed the utmost TA (0.74% and 0.78%, respectively, in NEha and NEh10a) greater than those of other groups (0.55%–0.63%, *p* < .05), which is in line with the minimum limit (0.6%) for yogurt recommended by Codex (Standard Codex, 2011). In general, the addition of microencapsulation and nanoencapsulation did not influence the pH range and acidity of the yogurt samples as there are some factors influencing the fermentation process and the growth of LAB.

**FIGURE 2:**
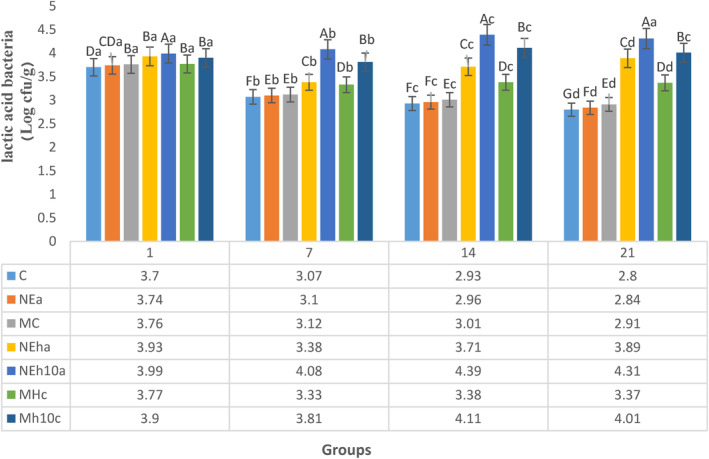
The trend of lactic acid bacteria at different fortified yogurts during the storage of yogurt samples. Small dissimilar scripts show significant differences (*α* = .05) each day (line). MC, CaCl_2_‐Microencapsules Yogurts; Mh10C, CaCl_2_‐microencapsulated‐FPH yogurt containing less than 10 kDa protein; MhC, CaCl_2_‐Microencapsulated‐FPH yogurt containing more than 10 kDa protein; NEa, Alg‐Na nanoemulsion Yogurts; NEh10a, Alg‐Na nanoemulsion‐FPH yogurt containing less than 10 kDa protein; NEha, Alg‐Na nanoemulsion‐FPH yogurt containing more than 10 kDa protein. The differences between the means with different letters in the same column are significant (*p* < 0.05).

The trend of LAB of yogurt samples at the different controlled times was plotted in Figure 2. LAB value was reduced from 3.70 to 2.80 log CFU/mL in control samples at the refrigerated storage with the same pattern in the MC and NEa groups (*p* > .05) in yogurts without FPH. The greatest LAB viable count observed in yogurts fortified with FPH, NEh10a, and Mh10C on day 14 considerably (*p* < .05) was more significant than those of control and other yogurts even yogurts fortified with FPH, including NEha and MhC. Among the fortified yogurts, the maximum reduction of LAB rate observed in groups without FPH such as NEa (2.84–3.11 log CFU/mL) in a time‐dependent manner of refrigerated storage after MhC, MC, and control groups (*p* > .05) at the same pattern. Despite the number of LAB in the FPH yogurts considerably (*p* < .05) more than the amount of the control and non‐FPH groups (Figure 2), surprisingly, the acidity of the FPH groups decreased and significantly became lower than the afore‐mentioned yogurts (Table 3).

#### 3.2.2 Syneresis

The syneresis result of the fortified yogurts is expressed in Table 5. Free whey of yogurt samples increased in a time‐dependent manner during cold storage while WHC decreased. The relation tendency of ejected whey (syneresis) and viscosity in all models was exhibited to be inversely proportional. Out of the groups, yogurt fortified with different types of FPH decreased syneresis and increased WHC, particularly in microencapsulation. All the yogurt samples showed less syneresis than those of the control (*p* < .05). The least values of syneresis observed in Mh10C containing FPH less than 10 kDa on days 1 (7.47%), 7 (9.43%), 14 (10.70%), and 21 (12.80%) without any considerable changes (*p* > .05) in comparison with MhC and MC (exception on day 21).

**TABLE 5:** The between‐subject effects of the two‐way ANOVA test for the interaction effect of independent variables of different fortified yogurts, and sampling days on total count, sample's yeast, and coliform.

Effect	P.E.Sq	*F*	Sig.
TPC	0.980	151.40	.000
Coliform	0.754	9.53	.000
Yeast	0.856	18.52	.000

Abbreviations: P.E.Sq, Partial Eta square; TC, Total count.

The data associated with viscosity, WHC, and syneresis criteria of yogurt samples under the different treatments in refrigerated storage are shown in Table 5. Accordingly, the two‐way ANOVA was significant (*p* < 001) for the main effects (not given in the Tables), including the treatment and refrigeration time. The difference means due to the interaction between the aforementioned independent variables was important (*p* < .05).

#### 3.2.3 Viscosity and water‐holding capacity

**FIGURE 3:**
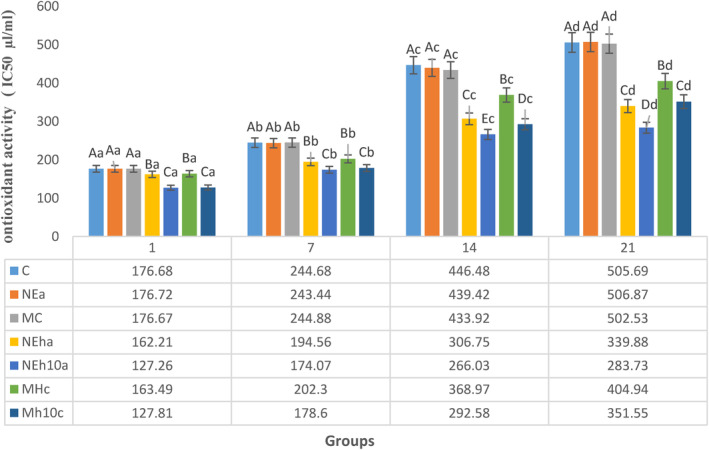
The trend of antioxidant activity of mean FRAP (mg/g) was affected by the differently treated fortified yogurt on the sampling days. C, Control; MC, CaCl_2_‐Microencapsules Yogurts; Mh10C, CaCl_2_‐Microencapsulated‐FPH yogurt containing less than 10 kDa protein; MhC, CaCl_2_‐Microencapsulated‐FPH yogurt containing more than 10 kDa protein; NEa, Alg‐Na nanoemulsion Yogurts; NEh10a, Alg‐Na nanoemulsion‐FPH yogurt containing less than 10 kDa protein; NEha, Alg‐Na nanoemulsion‐FPH yogurt containing more than 10 kDa protein. The differences between the means with different letters in the same column are significant (*p* < 0.05).

The apparent viscosity and WHC of yogurts were assayed to exhibit possible alteration in the quality of samples within 21 days of cold storage. The results (Figure 3) directed that the FPH <10 kDa performed yogurt with the most robust gel network after 21 days, followed by the FPH yogurt >10 kDa in group NEha. On day 7, FPH of *Planiliza abu* caused greater viscosity in associated yogurts with no significant difference compared with MC (*p* > .05). Generally, NEha could not show a suitable viscosity of yogurt compared with MhC (Table 6) might be due to less hydrophilic activity in higher weight peptides. Water‐holding capacity and viscosity of yogurt samples decreased in a time‐dependent manner (Table 7), with the least and greatest ranges of WHC observed in control (81.6%–88.5%) and NEh10a (87.2%–92.5%), respectively, during the cold storage. Table 6 shows that WHC of Mh10C and NEh10a from day 1 did not considerably (*p* > .05) decrease till day 21. After a week of cold storage, the greatest WHC (90.5%) was observed in NEh10a without any considerable variation (*p* > .05) in comparison with MC but higher than other treatments and control (*p* < .05). On day 21, the greatest WHC was observed in NEh10a (87.2%) with no significant changes compared with NEha, but significantly (*p* < .05) greater against other groups (Table 8). Non‐FPH yogurt samples and control, therefore, resulted in a weaker texture for the associated yogurt samples. Nevertheless, yogurt with low or free fat shows weak uniformity and stability, resulting in a weak gel, declining WHC, and phase separating. Generally, whole milk usage showed higher viscidity of concerning yogurt, which can be because of the more significant total solids of the milk resulting in the firmness of yogurt gel. Textural and matrix formation of yogurt is influenced by protein availability. So, it makes sense that FPH provided protein during the textural formation thus improving the matrix strength and viscosity.

**TABLE 6:** Estimated marginal means of collected count (log CFU/mL), coliform (log CFU/mL), and yeast (log CFU/mL) at different fortified yogurts during the cold storage of yogurt.

	d	C	MC	Mh10C	MhC	NEa	NEh10a	NEha
TPC	1	6.31 ± 0.009^a^	6.32 ± 0.009^a^	6.32 ± 0.009^a^	6.32 ± 0.009^a^	6.31 ± 0.009^a^	6.31 ± 0.009^a^	6.31 ± 0.009^a^
	7	4.84 ± 0.009^bA^	6.96 ± 0.009^bB^	7.22 ± 0.009^bC^	7.09 ± 0.009^bB^	6.96 ± 0.009^bB^	7.38 ± 0.009^bC^	7.29 ± 0.009^bC^
	14	6.24 ± 0.009^a^	6.29 ± 0.009^a^	6.36 ± 0.009^a^	6.36 ± 0.009^a^	6.25 ± 0.009^a^	6.41 ± 0.009^a^	6.36 ± 0.009^a^
	21	4.81 ± 0.009^bA^	4.92 ± 0.009^cA^	5.27 ± 0.009^cB^	5.13 ± 0.009^cB^	4.90 ± 0.009^cA^	5.44 ± 0.009^cB^	5.33 ± 0.009^cB^
Clf	1	ND	ND	ND	ND	ND	ND	ND
	7	ND	ND	ND	ND	ND	ND	ND
	14	ND	ND	ND	ND	ND	ND	ND
	21	ND	ND	ND	ND	ND	ND	ND
Yst	1	ND	ND	ND	ND	ND	ND	ND
	7	ND	ND	ND	ND	ND	ND	ND
	14	1.12 ± 0.01^a^	1.15 ± 0.01^a^	ND	ND	1.19 ± 0.01^a^	ND	ND
	21	1.98 ± 0.01^bA^	1.84 ± 0.01^bA^	0.95 ± 0.01^B^	1.25 ± 0.01^C^	1.94 ± 0.01^bA^	0.99 ± 0.01^B^	1.34 ± 0.01^C^

*Note*: The differences between the means with different letters in the same column are significant (*p* < 0.05).

Abbreviations: Clf, Coliforms; MC, CaCl_2_‐Microencapsules Yogurts; Mh10C, CaCl_2_‐Microencapsulated‐FPH yogurt containing less than 10 kDa protein; MhC, CaCl_2_‐Microencapsulated‐FPH yogurt containing more than 10 kDa protein; ND, not defined; NEa, Alg‐Na nanoemulsion Yogurts; NEh10a, Alg‐Na nanoemulsion‐FPH yogurt containing less than 10 kDa protein; NEha, Alg‐Na nanoemulsion‐FPH yogurt containing more than 10 kDa protein; TC, Total count; Yst, Yeast and molds.

**TABLE 7:** The between‐subject effects for the interaction effect of independent variables of different fortified yogurts, and sampling days on viscosity, water‐holding capacity, syneresis, antioxidant activity, and ferric reducing antioxidant power.

Effect	P. E.Sq	*F*	Sig.
Viscosity	0.968	93.78	.000
Water‐holding capacity	0.872	21.19	.0000
Syneresis	0.872	21.19	.000
Antioxidant activity	0.996	69.91	.000
FRAP	0.919	35.29	.000

Abbreviations: FRAP, ferric reducing antioxidant power; P.E.Sq, Partial Eta square.

**TABLE 8:** Estimated marginal means of viscosity (cps), water‐holding capacity (%), and syneresis (%) at different fortified yogurts and sampling days.

	D	C	MC	Mh10C	MhC	NEa	NEh10a	NEha
V	1	4042.0 ± 4.3^aA^	4289.3 ± 4.3^aB^	4257.3 ± 4.3^aC^	4270.0 ± 4.3^aC^	4166.3 ± 4.3^aD^	4207.3 ± 4.3^aE^	4187.3 ± 4.3^aD^
	7	3909.7 ± 4.3^bA^	4216.0 ± 4.3^bB^	4216.6 ± 4.3^bB^	4210.0 ± 4.3^bB^	4066.6 ± 4.3^bC^	4203.0 ± 4.3^aB^	4138.6 ± 4.3^bD^
	14	3784.0 ± 4.3^cA^	4131.6 ± 4.3^cB^	4169.3 ± 4.3^cC^	4137.0 ± 4.3^cB^	3959.3 ± 4.3^cD^	4187.0 ± 4.3^bC^	4079.0 ± 4.3^cD^
	21	3716.0 ± 4.3^dA^	4079.3 ± 4.3^dB^	4140.3 ± 4.3^dC^	4102.3 ± 4.3^dD^	3904.6 ± 4.3^dE^	4166.3 ± 4.3^cF^	4046.6 ± 4.3^dB^
WHC	1	88.5 ± 0.07^aA^	91.9 ± 0.07^aA^	90.0 ± 0.07^aA^	89.8 ± 0.07^aA^	89.6 ± 0.07^aA^	92.5 ± 0.07^aA^	92.5 ± 0.07^aA^
	7	86.2 ± 0.07^bA^	89.7 ± 0.07^bB^	88.1 ± 0.07^bC^	87.8 ± 0.07^bC^	87.6 ± 0.07^bC^	90.5 ± 0.07^bB^	90.2 ± 0.07^bB^
	14	84.3 ± 0.07^cA^	87.9 ± 0.07^cB^	86.7 ± 0.07^cC^	86.0 ± 0.07^cC^	85.5 ± 0.07^cC^	89.3 ± 0.07^bB^	88.4 ± 0.07^cB^
	21	81.6 ± 0.07^dA^	85.2 ± 0.07^dB^	84.8 ± 0.07^dB^	84.6 ± 0.07^dB^	83.9 ± 0.07^dB^	87.2 ± 0.07^cC^	86.2 ± 0.07^dBC^
Sy	1	11.46 ± 0.07^aA^	8.05 ± 0.07^aB^	7.47 ± 0.07^aB^	7.50 ± 0.07^aB^	10.38 ± 0.07^aA^	9.99 ± 0.07^aAB^	10.17 ± 0.07^aA^
	7	13.73 ± 0.07^bA^	10.27 ± 0.07^bB^	9.43 ± 0.07^bB^	9.73 ± 0.07^bB^	12.32 ± 0.07^bAB^	11.8 ± 0.07^bAB^	12.2 ± 0.07^bAB^
	14	15.63 ± 0.07^cA^	12.09 ± 0.07^cB^	10.70 ± 0.07^cC^	11.52 ± 0.07^cB^	14.50 ± 0.07^cAB^	13.30 ± 0.07^cB^	14.00 ± 0.07^cAB^
	21	18.31 ± 0.07^dA^	14.75 ± 0.07^dB^	12.80 ± 0.07^dC^	13.8 ± 0.07^dBC^	16.05 ± 0.07^dB^	15.15 ± 0.07^dB^	15.37 ± 0.07^dB^

*Note*: The differences between the means with different letters in the same column are significant (*p* < 0.05).

Abbreviations: C, Control; D, days; MC, CaCl_2_‐Microencapsules Yogurts; Mh10C, CaCl_2_‐Microencapsulated‐FPH yogurt containing less than 10 kDa protein; MhC, CaCl_2_‐Microencapsulated‐FPH yogurt containing more than 10 kDa protein; NEa, Alg‐Na nanoemulsion Yogurts; NEh10a, Alg‐Na nanoemulsion‐FPH yogurt containing less than 10 kDa protein; NEha, Alg‐Na nanoemulsion‐FPH yogurt containing more than 10 kDa protein; Sy, Syneresis; V, viscosity; WHC, water‐holding capacity.

In this study, the viscosity of all samples decreased in a time‐dependent manner, except for NEh10a yogurt samples showed no considerable discrepancy (*p* > .05) during 7 days of cold storage. Generally, the results of this study showed that FPH had good effectiveness on yogurt viscosity compared with control samples (*p* < .05) but FPH < 10 kDa caused higher viscosity of yogurt compared with FPH > 10 kDa.

### 3.3 Microbial analysis

The data regarding the TPC, coliforms, and yeasts of yogurt samples under the different treatments during the refrigerated storage are shown in Table 7. Accordingly, the ANOVA was highly significant (*p* < 001) for the treatment, refrigeration period, and interaction (Table 4). The two‐way ANOVA (Table 4) outcomes showed that not only the interaction effect of various fortified yogurts on total plate count (TPC), coliform count, and yeast count was significant (*p* < .05) on each sampling day of the experiment but also the main effect (not given in the Tables) of each afore‐mentioned independent variable was significant (*p* < .05). However, the interplay result of independent variables is shown in Table 7.

#### 3.3.1 Total plate count (TPC)

The results showed a quadratic trend of TPC for each treatment over time (Table 8) so that the utmost and the minimum TPC observed on days 7 and 21, respectively, against the control count, showed the maximum TPC observed on the first day of refrigeration and significantly reduced up to day 7. Henceforth, The TPC of treated yogurts gradually decreased, ranging from 4.9 to 5.4 log CFU/mL on day 21. The first‐day TPC of control was 6.3 logs CFU/mL and demonstrated no considerable difference (*p* > .05) in comparison to other groups. The maximum TPC was performed on day 7 in NEh10a (7.38 log CFU/mL) with no importance to other FPH groups, NEha and Mh10C. At the same time, the minimum TPC of treated yogurts was observed in MC and NEa (6.9 logs CFU/mL) considerably (*p* < .05) greater than the control (4.84 logs CFU/mL). The sudden increase in TPC could be interpretably due to the effect of fish peptides on the growth of bacteria, similar to the results of LAB (Figure 2).

#### 3.3.2 Coliform

According to the outcomes (Table 8), the microbial study of the yogurt groups showed no presence of coliform bacteria during refrigeration. This might be due to the great hygienic measures in the experiment that prohibited postmanufacture spoilage as the chief issues for coliforms increase in yogurt production are microbiological considerations. Some germs may be introduced into yogurt after the initial milk is treated with heat.

#### 3.3.3 Yeast and molds

The count of yeast and molds of the various fortified yogurts is expressed in Table 8. The data did not find units for yeast and mold in the fresh yogurt, which could be because of the sanitary achievement in this experiment. The appearance of yeast and molds in yogurts was not detected by 14‐day refrigeration of this experiment, while it was 1.1 logs CFU/mL in control and non‐FPH yogurts (*p* > .05) on day 14 but not defined (ND) in yogurts fortified with FHP at the same time (Table 8).

On day 21, the maximum value of grown mold and yeast was observed in control (1.98 log CFU/mL) with no significant difference against non‐FPH yogurts, including MC and NEa (Table 8). Simultaneously, the minimum value of yeast and mold was 0.95 log CFU/mL in Mh10C with no remarkable variation (*p* > .05) compared with that of NEh10a followed by the value of MhC (1.2 log CFU/mL) with no remarkable variation (*p* > .05) against the value of NEha (Table 8). Total yeast and mold counts recommended are less than 10 CFU/g in yogurts (Kiros et al., 2016).

### 3.4 Antioxidant activity

**FIGURE 4:**
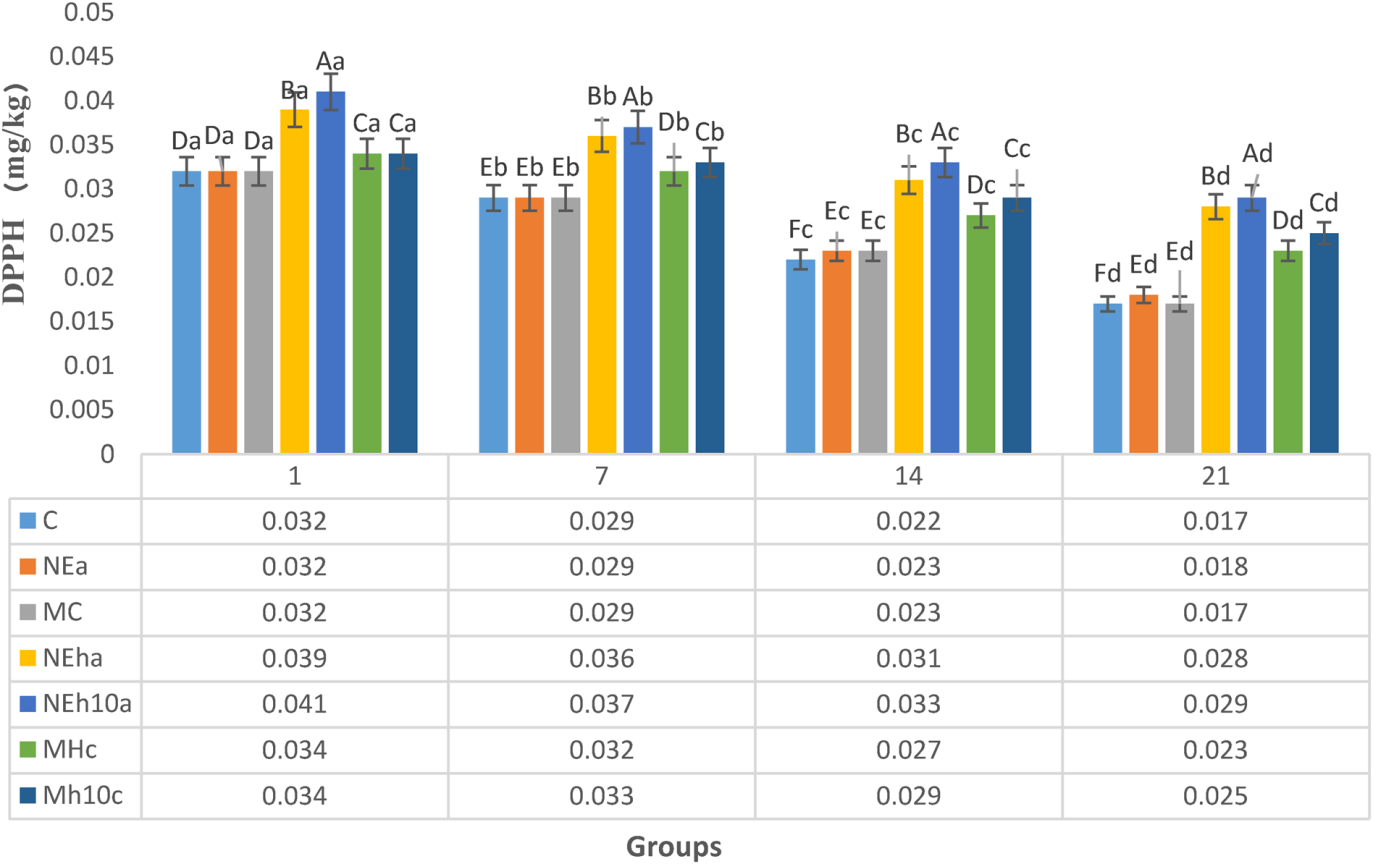
The trend of DPPH value (IC50 μL/mL) was affected by the differently treated fortified yogurt on the sampling days. C, Control; IC50 (μL/mL), Inhibitory concentration at which 50% of DPPH radical is scavenged; MC, CaCl_2_‐Microencapsules Yogurts; Mh10C, CaCl_2_‐Microencapsulated‐FPH yogurt containing less than 10 kDa protein; MhC, CaCl_2_‐Microencapsulated‐FPH yogurt containing more than 10 kDa protein; NEa, Alg‐Na nanoemulsion Yogurts; NEh10a, Alg‐Na nanoemulsion‐FPH yogurt containing less than 10 kDa protein; NEha, Alg‐Na nanoemulsion‐FPH yogurt containing more than 10 kDa protein. The differences between the means with different letters in the same column are significant (*p* < 0.05).

Antioxidant compositions of yogurts are associated with eliminating free radicals, resulting in increased shelf life, and postponing the process of lipid oxidation. The antioxidant potential of FPH‐fortified yogurt was measured using each yogurt sample supernatant obtained during filtration and centrifugation. Outcomes of the two antioxidant evaluations, containing FRAP, that, ferric reducing antioxidant power assays and DPPH radical scavenging assays, are depicted in Figures 3 and 4. The highest FRAP was observed in group NEh10a (0.41 mg/g) without any considerable difference (*p* > .05) in comparison with NEha (0.40 mg/g) which was the lowest than other treatments or control. This superiority of FRAP of NEh10a or NEha groups continued to exist through cold storage, which could be necessary for the yogurt oxidative stability (Figure 3). The FRAP of the control group exhibited the least values during the sampling days without any considerable difference (*p* > .05) in comparison with MC, and NEa, which confirmed that FPH‐fortified yogurt with more than 10 kDa peptides has not any extra antioxidant activity in comparison with non‐FPH‐fortified yogurt (Figure 3) because the composition and availability of amino acids in each formula are different, thus affecting the antioxidant.

DPPH is a stable nitrogen‐based free radical which receives hydrogen or an electron to get a stable diamagnetic molecule. Figure 3 depicts DPPH radical scavenging activities of different yogurt samples. As it was anticipated, the lower DPPH value was seen in the FPH yogurt containing less than 10 kDa protein, including NEh10a and Mh10C groups. The higher DPPH quantity means the lower antioxidant activity presented in the yogurt samples. No considerable change (*p* > .05) was seen among non‐FPH yogurts and the control during the cold storage (Figure 4) concerning the DPPH. For each yogurt sample, the difference between the DPPH values on days 1 and 7 was negligible. However, this difference significantly increased after the first week of the cold storage and showed greater ones on day 14.

### 3.5 Sensory evaluation

**FIGURE 5:**
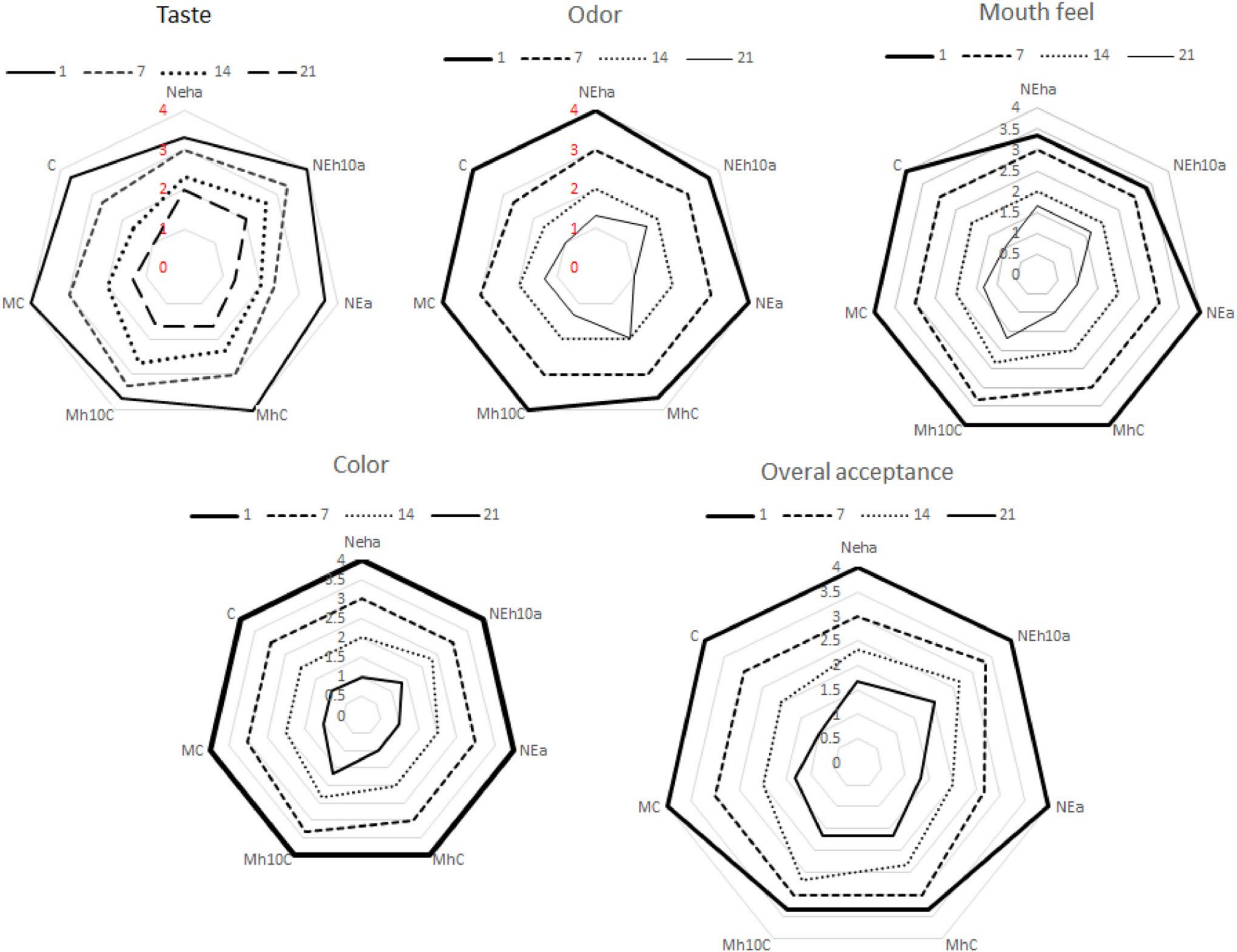
The situation of sensory assessment of yogurt samples. MC, Yogurts fortified with CaCl_2_‐Microencapsules; Mh10C, yogurt of less than 10 kDa‐FPH CaCl_2_‐Microencapsulated yogurt; MhC, yogurt of more than 10 kDa‐FPH fortified with CaCl_2_‐Microencapsules; NEa, Yogurts fortified with Alg‐Na nanoemulsion; NEh10C, yogurt of less than 10 kDa‐FPH fortified with Alg‐Na nanoemulsion; NEha, yogurt of more than 10 kDa‐FPH fortified with Alg‐Na nanoemulsion.

The average numbers of sensory features of the yogurt models are depicted in Figure 3. Regarding the taste, the highest mean score was 4 in groups NEh10a, MhC, and MC on day 1. After a week, the most incredible score was given to taste 3.33 in NEh10a with no considerable discrepancy (*p* > .05) in comparison with Mh10C (3.33). The taste score was decreased in a time‐dependent manner and reached 2.67 in the same groups on day 14 (Figure 5). The odor scores of all yogurt samples of treatments were 4.0, 3.0, and 2.0 on days 1, 7, and 14, with negligible differences (*p* > .05). A slight fishy smell was detected in Mh10C and NEha (Figure 5) after a day of cold storage, but all yogurt had a better smell compared with the control on day 7, similar to the condition that occurred on day 14. However, on this day, the odor of yogurt‐treated samples was accepted as moderate (relatively off‐odor), which was slightly more than the control. The scores of “mouth feel” for all groups were 4.0, except for Mh10C and MhC (3.33 and 3.33) on day 1. After a week, the most outstanding mean score of “mouth feel” was 3.33, observed in NEh10a, slightly more than other groups (3.0, *p* > .05). This score was highest in NEh10a after 2 weeks (2.3) without any considerable change (*p* > .05) in comparison with other groups (2.0). The outcomes revealed that the NEh10a yogurt has a good mouth feeling for panelists compared with different yogurt samples. On day 1, all groups showed the maximum mean score (4.0) for the color of yogurt samples, except for Mh10C (3.33) and MhC (3.33). On days 7 and 14 of cold storage, the mean scores of “color” of yogurt samples were the same as the scores of “mouth feel.” The overall acceptance attribute was the least (3.33) in yogurt samples, Mh10C and MhC less than other groups (*p* < .05) presented the maximum mean score (4.0). On day 7, the overall acceptance attribute showed the highest mean score in NEh10a yogurt samples (3.33) with a meaningful difference (*p* > .05) compared with NEa yogurt samples and control (2.67). On day 14, the most significant overall acceptance criteria were observed in groups NEh10a and Mh10C (Figure 5). In the present study, the nanoemulsion and microcapsule of FPH added to plain yogurt did not contain any flavoring compounds.

## 4 DISCUSSION

Similar to this study (Figure 1), some FPHs have a relatively high content of free amino acids like glycine and alanine that makes them taste sweeter (Norziah & Ching, 2000). The fish protein analysis (Table 2) showed a close content of *Planiliza abu* amino acid profile to the hen egg (Macelline et al., 2021). At the beginning of this study, pH and TA were in line with the acceptable consumer value (pH 4.4–4.6) recommended for yogurt (Benedetti et al., 2016; Frye & Kilara, 2015). Similar to this trial, the pH of yogurt enriched with microencapsulated Stripped Weakfish (*Cynoscion guatucupa*) protein hydrolysate reached 4.3 after a week (Lima et al., 2021).

These results showed yogurt fortified with NE‐FPH had extra effects on TA formation. The variation of acidification among the different fortified yogurts occurs due to the differences in the quantities of viable microbes and fermentation of lactose to lactic acid (Sigdel et al., 2018; Tseng & Zhao, 2013; Vandera et al., 2019), which could be because of the effect of FPH particularly in small peptides less than 10KDa (Figure 1).

Folic acid‐supplemented yogurt (Aryana, 2003) showed two times greater values of TA (1.3%) in comparison with yogurt samples after 4 days of refrigeration. Like the present project (Table 3), Lima et al. (2021) presented that the addition of FPH in the form of free or microencapsulate increasingly influences the TA of yogurt after a week of cold storage (1.08–1.09, *p* > .05). Still, Afzaal et al. (2018) showed that microencapsulated FPH promoted TA production (0.63%) significantly greater than the free form of FPH in yogurt. Therefore, supplemented FPH may have enhanced bacterial growth in fortified yogurts and amplified the acidifying capability of the microbial activities (Sah et al., 2015) due to enzymatic hydrolysis. It creates no harm in amino acid formulas of proteins, rather it causes the breaking of the proteins' peptide bonds. The amino acids or low‐weight peptides produced in this hydrolysis process are possible to be applied as nitrogen sources to help the development of many bacteria. Fish collagen‐derived bioactive peptides did not change the PH (4.5) and TA (0.82) of yogurts among the different concentrations after 14 days (Ayati et al., 2022), which were greater than those of this study showed lower TA (0.6–0.7%) and pH (around 4.2). They recommended that the significant alterations in these subjects might be due to the low buffering properties of the fish collagen‐derived bioactive peptides applied in the yogurt. The span of acceptable pH range of yogurts entering the markets is endorsed to be 3.27–4.59 during 14 days (Cho et al., 2020). After 21 days, the TA of the all‐fortified yogurt samples was above the Codex minimum level of TA.

There is no doubt that lactic acid bacteria do not have appropriate potential to hydrolyze fish protein to amino acids. On the other hand, the amino acids or small molecular peptides, which were produced through the hydrolysis process, may be applied as nitrogen sources to promote the development of starter strains (du Toit et al., 2011) as well as casein hydrolysates (Ma et al., 2019). Likewise, yogurt supplemented with FPH, particularly in the form of nanoemulsion (NEha and NEh10a), thus, significantly promoted the growth of LAB (Figure 2).

The difference associated with LAB count in different groups might be due to the chemical stability of NE and ME structure regarding slow release and availability of their contents to LAB (El‐Sayed et al., 2022) or the buffering property of peptides (Ayati et al., 2022). This assumption is supported by less viable cell counts of LAB in yogurts without FPH (Figure 2). The count of LAB of some studied yogurts spanned from 8.36 to 8.71 log CFU/mL (Atallah et al., 2020). The mentioned quantities are more than those of this study that could be due to the value of the microbial count of starter added into tested yogurts.

Another study reported that the maximum TPC of yogurt enriched with encapsulation was 6.92 log CFU/mL after 2 weeks, slightly greater than those of this study (6.3 log CFU/ mL) conducted with microencapsulated yogurt at the same time, and the reason could be the higher acid production and the applied starter volume (Afzaal et al., 2018). With the increase of coconut from 10% to 30% added to yogurt, the TPC decreased (Ndife et al., 2014). However, the highest TPC was 5.9 log CFU/mL on day 1 (Table 5), which showed less value than those of all fortified yogurts reported in this study. Based on another finding (S Abdel‐Ghany & A Zaki, 2018), the TPC was enhanced from 8.0 log CFU/mL on day 1 to 8.6 log CFU/mL on day 12, while the yogurt amplified with 5% date syrup and 10% bovine colostrum against control showed an increase from 8.1 to 8.7 log CFU/mL. They claimed that this further bacterial growth might be due to the various effects of several compounds added to yogurt. On the other hand, the TPC of different yogurt samples fortified with psyllium husk (Plantago ovata) was 5.7 log CFU/mL in the control with remarkable differences with treated yogurts showing an increase in a dose‐dependent manner on day 7 at refrigerated condition (Bhat et al., 2018) which could have been because of the enhanced moisture content in the yogurt that is enriched by fiber.

Based on Canadian legislation (Ghalem & Zouaoui, 2013), the supplemented yogurts should have acceptable quality and not allow any risk of contamination for the users. Similar to the mentioned study (Table 5), coliform was not observed at any days of refrigeration in a study conducted on yogurt supplemented with date syrup and bovine colostrum at different concentrations (S Abdel‐Ghany & A Zaki, 2018).

In carrot powdered fortified yogurts, mold and yeast were observed at any concentration from day 7 at 5°C (5.2 log CFU/mL) without any meaningful difference (*p* > .05) compared with control (Madora et al., 2016). In research done on yogurt fortified with 5% date syrup and 10% bovine colostrum (S Abdel‐Ghany & A Zaki, 2018), yeast and molds were revealed after the first week of the experiment so that they ranged from 2.6 to 3 logs CFU/mL, respectively, on days 8 and 12 in treated yogurts against 2.9 to 3.04 log CFU/mL in the control at 5°C and the same time. Dissimilarly, in this study, the yeast and molds appeared after 2 weeks of refrigeration but were not defined in FPH‐fortified yogurts.

FPH has had an inhibitory role in the growth of yeast and mold (Table 5), which might be because of the part of low‐weight peptides with antimicrobial activity and the LAB in the preservation of the product, which is related to their ability to make lactic acid (Atallah et al., 2020). Yeast and mold appeared in yogurts fortified with co‐encapsulation of extra virgin olive oil nanoemulsion or nanocomposite and symbiotic bacteria (maltodextrin with *Lactobacillus acidophilus* and *Bifidobacterium bifidum*) on day 15 and increased on day 20 ranging from1.3 to 3.3 log CFU/mL (El‐Sayed et al., 2022), which was somewhat more remarkable than the results of this study at similar condition. Moreover, the occurrence of yeasts and molds in yogurt is evidence of the unacceptable hygienic conditions in production (Hervert et al., 2017).

Peptides in fish hydrolysates which are rich in hydrophilic amino acids might trap water and hence enhance the viscosity, similar to the yogurts fortified with soy showed two times more viscosity than the control (Drake et al., 2000). The lysine, glutamic, and aspartic acids, which can powerfully bind to water and cause WHC to increase compared with nonionizable polar groups (Gheshlaghi et al., 2021), were more remarkable contents in FPHs of *Planiliza abu* than other amino acids in this study (Table 2). The gradual decline different from another research (S Abdel‐Ghany & A Zaki, 2018) showed the viscosity or WHC increased within the time storage. The increase of viscosity and WHC of yogurts might be due to the binding of added protein (particularly in the nano form) with free water and reduction of flowability, involving the protein network and increasing the resistance of the sample to flow (Aziznia et al., 2008). Gelatin from sturgeon fish was used to reinforce the protein network, increase the WHC, and enhance the viscosity of yogurt (Gheshlaghi et al., 2021).

The syneresis values of the research (Table 7) on day 1 were 3–4 times less in yogurt provided with fish gelatin (Ma et al., 2019). The incremented trend of whey observed during the storage might be because of the differences in pH, acidity, and protein properties resulting in damage to the bonds with water (S Abdel‐Ghany & A Zaki, 2018).

Similar findings to this study (Figure 1) showed that the lower peptide fractions less than 10 kDa displayed higher ferric reducing properties (FRAP) than molecular peptides more than 10 kDa (Farvin et al., 2010). The 0.5% hydroponic ginseng extract‐fortified yogurt showed an FRAP value of 0.35 mM (Lee et al., 2021). The FPH with lower molecule weight donates the electron to free radicals and probably performs in yogurts. However, oxidative activities in yogurts can be eliminated or lessened (Nikoo et al., 2019). The FPH flavors are known to be necessary for rejecting yogurts (Murage et al., 2021). Thus, according to the panel ranks, the nanoemulsion FPH‐enriched yogurt will perhaps be accepted by more people, which suggests that there is a potential market for the FPH‐fortified yogurt, specifically between health‐sensible users. (Feizollahi et al., 2018). The masking effect of flavoring compounds, such as nanoemulsion and microcapsule has been described in some fortified foods (Kolanowski & Weißbrodt, 2007).

The finding of DPPH of this study was (Figure 2) similar to another research (Jónsdóttir et al., 2016) showed that higher electron‐donor properties of lower peptide fractions existing within yogurt storage are significant for the experimental ability to postpone oxidative changes. This indicates that peptides of these yogurt samples respond with free radicals to change them to firmer compounds. Similarly, other researchers (Farvin et al., 2010) displayed that the lower molecular weight fractions (3–10 kDa and < 3 kDa) remarkably have more radical scavenging properties compared with higher molecular weight peptides in yogurts.

According to sensory evaluation and panelist responses, NEh10a had a less fishy smell, more acceptable taste and mouth feel, and overall acceptance. It is concluded that yogurt fortified with FPH, particularly less than 10 kDa‐FPH, can enhance the level of yogurt to a desirable functional food with an appropriate gel network and consistency. Antioxidant activity and LAB survival showed excellent evaluation even on day 21.

## 5 CONCLUSION

In conclusion, our findings showed that the methanolic extract of D. sophia can be useful as an adjunctive treatment for hyperthyroidism, but further clinical trials are necessary to show exact benefits, especially considering the fact that the sample size of this study was not powered enough.

## AUTHOR CONTRIBUTIONS

**Maryam Ataee:** Methodology (lead); supervision (lead); writing – original draft (lead). **Nasrin Vakili:** Methodology, Writing (lead); review and editing (equal). **Shapour Kakoolaki:** Writing and review. **Hamed Ahari** Methodology (equal). **Arman Ghorbanzade:** Writing and review.

## CONFLICT OF INTEREST STATEMENT

The authors declare that they have no conflict of interest.

## ETHICS STATEMENT

This study involves human sensory analysis. No human ethics committee or formal documentation process is available and no coercion to participate, full disclosure of study requirements and risks, written or verbal consent of participants, and no release of participant data without their knowledge.

## Data Availability

The data sets generated during and/or analyzed during the current study are available from the corresponding author on reasonable request.
